# Dashboard Layout Effects on Drivers' Searching Performance and Heart Rate: Experimental Investigation and Prediction

**DOI:** 10.3389/fpubh.2022.813859

**Published:** 2022-02-14

**Authors:** Hao Yang, Yueran Wang, Ruoyu Jia

**Affiliations:** ^1^College of Mechanical and Material Engineering, North China University of Technology, Beijing, China; ^2^School of International Art Education, Tianjin Academy of Fine Arts, Tianjin, China

**Keywords:** physiological behavior, heart rate, eye movement, long short-term memory model (LSTM), shared car

## Abstract

Carsharing scale has been increasing rapidly with sharing economy. However, many users are reluctant to rent cars any longer due to the low-quality of interactive experience and usability, especially in terms of the dashboard design. This challenge should be urgently addressed in order to maintain the sustainable development of car-sharing industry and its environmental benefits. This study aims to investigate the relationship between users' driving activities (e.g., searching time, reading time, eye movement, heart rate) and dashboard layout. This study was conducted based on the experimental investigation among 58 respondents who were required to complete driving tasks in four types of cars with different dashboard layouts. Afterwards, a prediction model was developed to predict users heart rate (HR) based on the long short-term memory model, and logistic models were used to examine the relationship between the occurrence probability of minimum HR and dashboard reading. The results showed that the system usability of a dashboard was related to the drivers' eye movement characteristics including fixation duration, fixation times and pupil diameter. Most indicators had significant effects (*p* < 0.05) on the system usability score of corresponding dashboard. The long short-term memory model network (RMSE = 1.105, MAE = 0.009) was capable of predicting heart rate (HR) that happened in the process of instrument reading, which presented a periodic pattern rather than a continuous increase or decrease. It reflected that the network could better fit the non-linear and time-sequential laws of HR data. Furthermore, the probability of the lowest heart rate occurrence during the interaction with four dashboards was influenced by the average searching time, reading time and reading accuracy that were related to a specific layout. Overall, this study provided a theoretical reference for uncovering users' adaptive behaviors with the central control screen and for the optimal choice of a suitable dashboard layout in interface design.

## Introduction

Sharing economy has promoted automobile industry development, along with which car-sharing scale has been expanding dramatically. It is estimated that there have been more than five million carsharing users all over the world (1). However, many users are unwilling to use shared cars any longer, whilst the business modes (e.g., business to customer, peer-to-peer) are mature and friendly and carsharing has a series of environmental benefits (the reduction of vehicle ownership and emissions, the increase of flexibility of transit and the increase in land effectiveness) (2). To uncover the reason making these people abandon carsharing service is urgent and meaningful to maintain carsharing industry and promote its environmental benefits for sustainable development. Using behavior difference from the private cars is one of the key factors, where users can only own the right to use the car for a temporarily short term so that people will feel difficult in adapting themselves fully to shared cars, similar with the driving experience of a newly purchased car. Besides, car rental needs the users to go through processes of car ordering, car searching in the parking lot and getting in the car. When the user accesses to the car, it is necessary to judge whether the car is consistent with that displayed on the ordering APP interface and whether there are damaged or missing parts. These early cognitive activities would enhance users' sense of tension and fatigue, which is different from private cars that can be driven directly. It indicates that carsharing needs to be additionally compensated in terms of drivers' cognition to make the industry obtain more acceptance. In addition, for both business to customer car sharing or time-sharing car rental, human machine interface (HMI) features exclusive to an individual automobile brand are not suited for car sharing and will cut down the flexibility of users when interacting with the cars (3).

During the use of shared cars, drivers are required to read necessary information from dashboards to master running state of cars. This process indicates the significance of dashboard design for improving people's interactive experience with cars. In particular, the development of dashboard design has experienced many stages such as pure machinery, liquid crystal display (LCD) combined with machinery, and digital instrument. These different dashboard features may lead users to be exposed to different types of automobiles and a variety of dashboards in shared cars. As a consequence, the adaptability of dashboard needs to be revealed. No matter how advanced the dashboard display technology is, if the information recognition and visual interaction with the interface are improper, there will be increasing possibilities of safety issues. In particular, it is found that an off-road glance of more than 2 s would greatly increase the incidence of driving risks (4).

A digital instrument panel integrates more driving information into the interface, displaying navigation information, running status and vehicle driving control through an LCD screen. Drivers can get an overall description of the car intuitively from the instrument panel (5). However, the mode that drivers receive the feedback from cars, such as the visual feedback and auditory feedback, through digital instrument panels should be concerned, in particular when drivers should be informed appropriate feedback according to actual driving scenes. Nevertheless, the cognitive process of the use of shared cars are different from those of private cars (6). It is difficult for users to quickly understand and master the information through an unfamiliar interaction scheme. In the context of time-sharing rental cars, reading efficiency and mental load induced by different dashboard layouts are of great value to improve driving safety and user experience.

A driving simulator was therefore used to simulate acceleration, uniform velocity running and deceleration behaviors in this paper, in order to study the usability of different dashboard layouts. In particular, reading efficiency and driver's mental load of tested users were investigated with the requirements of reporting their readings on digital dashboard during driving. Afterwards, this study develops the prediction models of heart rates of drivers based both the long short-term memory model (LSTM) and logistic model in order to reveal the relationship between user heart rate and reading efficiency. Overall, this study is of significance to understand people's physiological behaviors toward environmental design in the shared cars and to promote the optimization of dashboard design.

## Literature Review

The HMI research works provided theoretical support for ergonomic assessment and interface evaluation of mechanical equipment and medical devices (7–9). The study on distraction and inattention caused by the HMI of in-vehicle information system (IVIS) was also a hot spot. For drivers, more than 90% information was obtained through visual channels when perceiving the external environment (10). In recent years, there were a growing number of driver assistance systems (DAS) in the automobile market. These functions, such as self-adaptive cruise and lane keeping system, reduced the measurement and control tasks of drivers (11), but put forward greater demands for the layout design works of dashboards and the recognition efficiency of drivers, especially in the era of aging. For example, from the perspective of take-over performance in intelligent vehicles, the average reacting time of older drivers was at least 1.2 s longer than that of young drivers (12). The in-vehicle HMI dimensions had a significant impact on drivers' task completion time (13), but a reasonable layout of the dashboard also played an important role in the driver's recognition efficiency. However, at present, people tended to just study the shape and character encoding of instrument panels (14). There are few researches on how these specific design factors are organized together to affect the driver, which may cause the influence of the design factors to be explained vaguely.

The display interface had an important effect on the visual load, work performance and subjective reaction in the use of automobiles (15). In the transition period to intelligent vehicles, new technologies drove the design works of automobile interactive experience to users' pleasure, integrated touch panel research, multi-channel interaction mode and so on (16). The emotion of car sharing users during driving was an important embodiment of the quality of in-vehicle display design. For example, drivers' satisfaction was related to the character lines of the dashboard with a 30% further converging point (17). For the automobile instrument panels, users could identify the images of dashboards according to the dimensions of visual acceptability, emotion and evaluation by the PAD scale, and there was a correlation between the arousal and the evaluation when reading the dashboard (18). A research using a virtual prototype also showed that a relationship existed between user impression and size, color, number of items, character/graphic size and gauge size of the dashboard (19). The results of these case studies illustrated that interaction performance of the dashboard could be adequately explained by design. However, in addition to impression and emotion, the effect of the designed dashboard layout on human physiological senses were not fully explained.

Automobile dashboards could be mainly divided into two types: center-locational ones and driver-orientational ones (20), and the visual ergonomic performance during interaction with the two types of panels was divergent. The dashboard system might produce different effects on the driver's psychological burden and feelings, thus affecting the information acquisition and operation behaviors of the driver in the cabin (21). The visual recognition efficiency of the form (circle or linear), indicators (pointers or bar graphs), and direction (horizontal or vertical) of the gauges on various instrument panels was different. Among these elements, the efficiency of linear instrument form with a pointer as the indicator and horizontal direction display was higher, while the drivers preferred round instruments subjectively (5).

For automobile users, the eye-movement data could be used to uncover their visual perception of grille, waistline, and engine hood, etc. (22). And some ocular parameters could distinguish a certain degree of sleepiness accurately in commercial motor vehicles (23). Besides, eye-movement interaction was an important interaction mode of autonomous driving (24), which was of great significance to the driving scenes with a large amount of information such as instrument reading. Eye-movement data, including gaze and blinks, could be combined with physiology signals (such as heart rate and skin conductance), facial expression and behaviors to evaluate the state of intelligent vehicle drivers effectively (25). Yet, some limitations existed in the collection and analysis of eye tracking data. For example, although the driver noticed a stimulus and some fixations occurred, the eye tracking information could not determine whether the fixations were due to interface interaction, or purely because the driver was obliged to look elsewhere (26). Therefore, this paper integrated task completion time and mental stress changes of the drivers with eye-movement analysis to get the conclusions.

Physiological control system maintained the balance between the system and the internal and external environment through the interaction and feedback among multiple variables. Among them, heart rate (HR), respiration (RESP), blood pressure (BP), and other important physiological variables could show complex variation patterns in different time scales (27). In transportation, such as cycling, electrocardiogram (ECG) could be used to estimate the real-time blood pressure as a feedback of the physiological signal changes during the process of behavior completion (28). For a driver who was stimulated by a nervous or intellectual signal while other conditions were relatively unchanged, the HR varied with the strength of the signal (29). On the part of car driving, the alteration of information input would give rise to the variation of stress level and then the fluctuation of HR, which was the physiological connection between HR and stress (30). Literatures (31–33) all applied ECG signals to analyze human-vehicle interaction behaviors. The measurement and analysis of driver ECG is a common research method from the field of neuropsychology, but from the perspective of driver visual attention, few studies combined ECG signal with eye movement. In this study, ECG data were put into practice to combine with the eye movement law and explore the subject's visual arousal degree during the dashboard reading experiment.

In recent years, data-driven prediction methods had attracted more attention from researchers (34–36). Like many machine learning applications, this kind of prediction problems adopted classification algorithm and regression algorithm to deal with the data. With a series of major breakthroughs in speech and behavior recognition in the field of transportation, the long short-term memory (LSTM) network showed a strong ability in information mining and deep representation when tackling time series problems. Duan et al. successfully solved the problem of pedestrian trajectory prediction using LSTM (37). And some scholars tried to apply this network to vehicle trajectory prediction (38, 39). Besides, Alahi et al. proposed a Social Long Short-Term Memory (S-LSTM) model (40). The space was meshed, and then the implicit features of nearby individuals around a certain one were pooled according to the grid structure. For people's mental stress prediction, the model could not only reflect the forward and backward correlation of HR signals, but also deal with the problem of long-term dependencies (41). However, research on the recognition of HR in the process of instrument reading is not sufficient. For the prediction of HR data during the specific task, the parameter adjustment of the model needs to be studied.

## Methods

### Experiment

**Figure 1:**
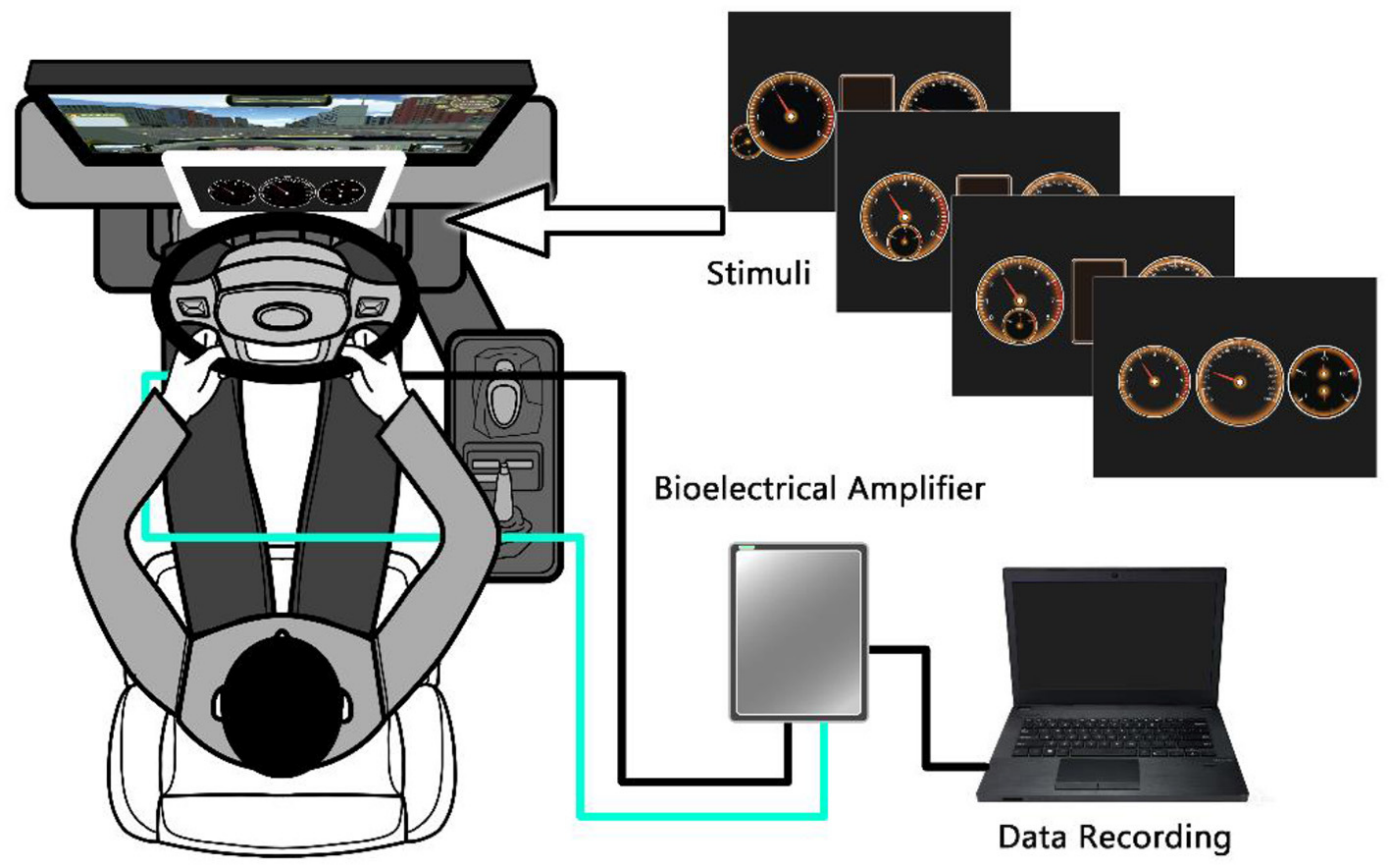
Diagram of experimental design.

The study was oriented to typical dashboard layouts and used a simple driving simulator to conduct experiments (Figure 1). The simulator was commonly used in driving schools in China, and existing research showed that it could effectively collect people's driving behaviors (13). Interaction efficiency, eye movement characteristics, mental stress changes and system usability were collected.

#### Stimuli

**Figure 2:**
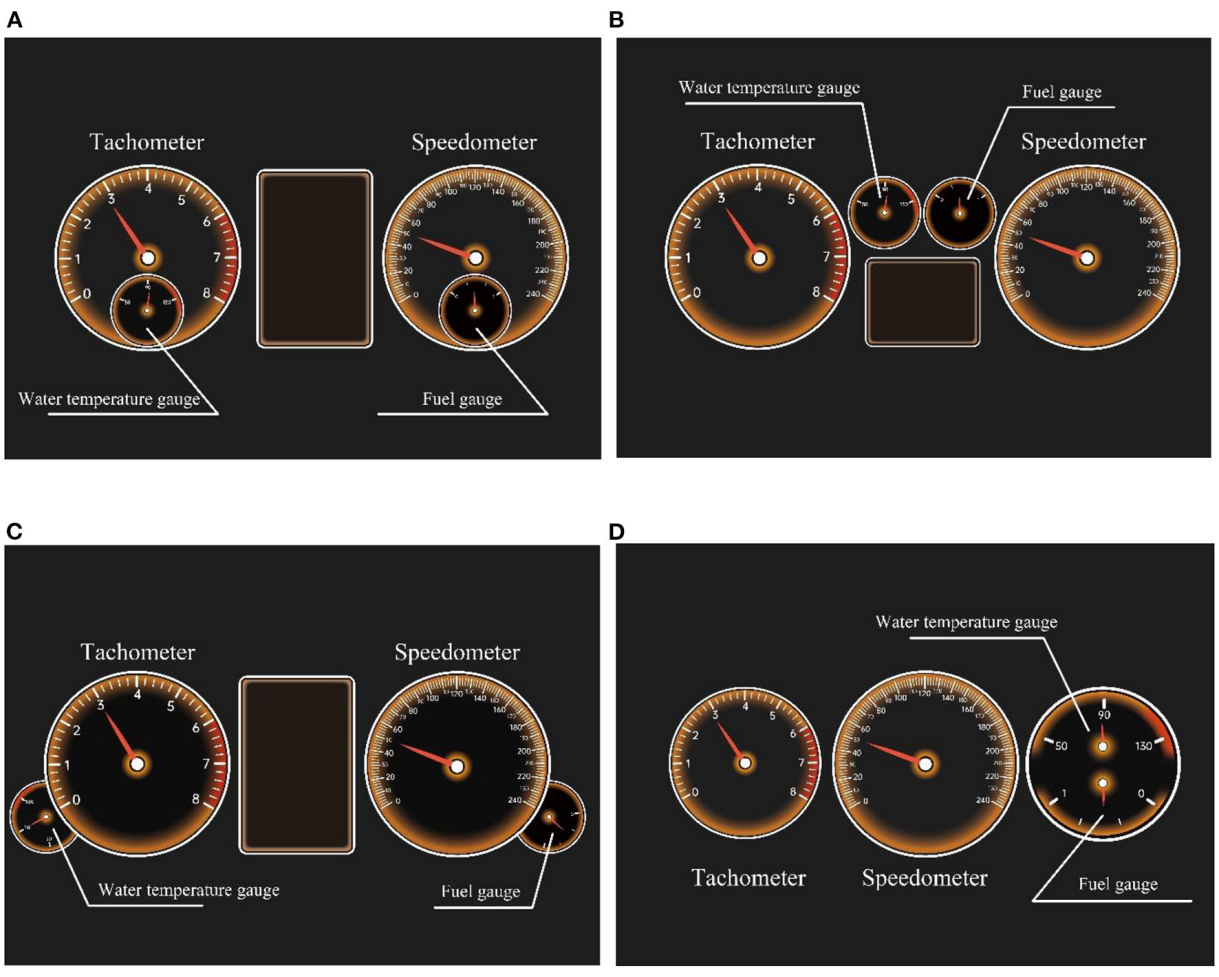
The stimuli of the experiment: **(A–D)** four types of dashboard layouts.

Digital dashboards of different automobile brands present various information element layouts, which are based on the distinctions of the instrument information architectures. Conventional functions of the dashboards are composed of relatively fixed parts, which can be summarized as: tachometer, speedometer, water temperature gauge, fuel gauge, gear information, signal light, air temperature, time, multimedia information and other driving related information. According to our investigation to the market and the conclusion of literatures (42, 43), the current layouts of automobile dashboards could be divided into four types (Figure 2).

Interface prototypes of four kinds of dashboard layouts as the stimuli were adopted in this paper. These prototypes were saved as GIF images and the pointers could rotate at a certain speed to simulate the readings of the dashboard. The dashboard size, line width, division value, scale and pointers were unified. The colors of the background, pointers and scale lines of the instrument panels were set to black, red and white, respectively. In order to avoid the difference of scale line density on arcuate meters from affecting the subjects' cognition, the instruments in the four interface prototypes were all circular. An iPad 2 (1024 ^*^ 768dpi) was used to present the prototypes, which was attached in front of the dashboard position of the driving simulator's screen (21.5 inches, 1920^*^1080dpi). The size of the prototypes on the iPad was the same as the dashboard in the simulator display, and other information on the screen would not be sheltered. The subjects were asked to use the driving simulator to complete the driving tasks.

#### Equipment

The ECG signals were collected by a 16-channel bioelectrical amplifier (Neuracle Co. Ltd., China) equipped with a NeuSen system. Two electrodes were attached to the back of the subjects' hands to gather the signals. The amplifier would not hamper the driver's operations. The sampling frequency was 1,000 Hz.

After analyzing the raw ECG with noise and observing the amplitude spectrum, a band-pass filter was devised by MATLAB ver. R2018a. By filtering the original ECG signals, high frequency noise and interferences were removed. The filtered signals including P waves, QRS waves, T waves and U waves were more in line with the rhythm of heartbeat.

For collecting the subjects' eye movement data, a head mounted eye tracker (SMI Co. Ltd., German) was utilized with a sampling frequency of 60 Hz.

#### Procedure

**Figure 3:**
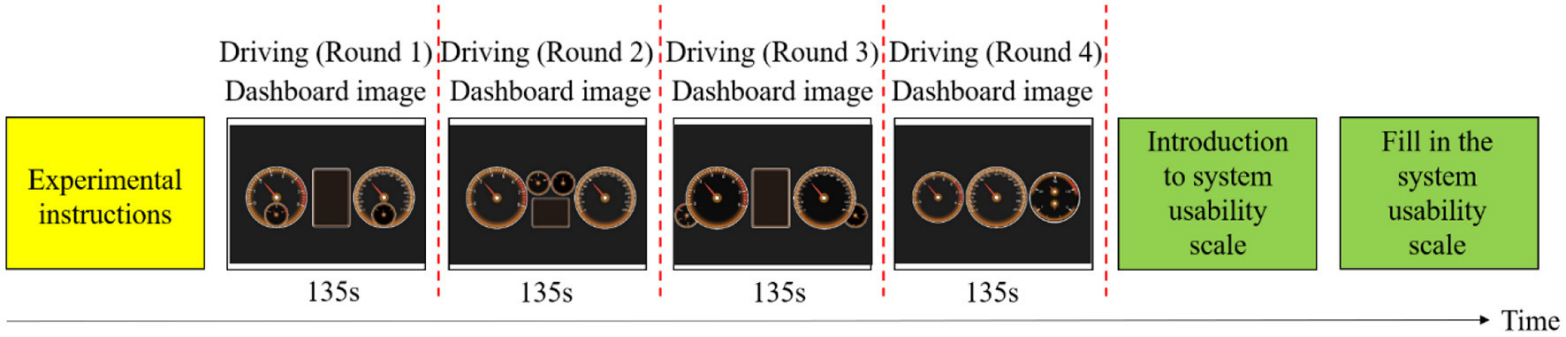
Workflow diagram of the experiment.

**Figure 4:**
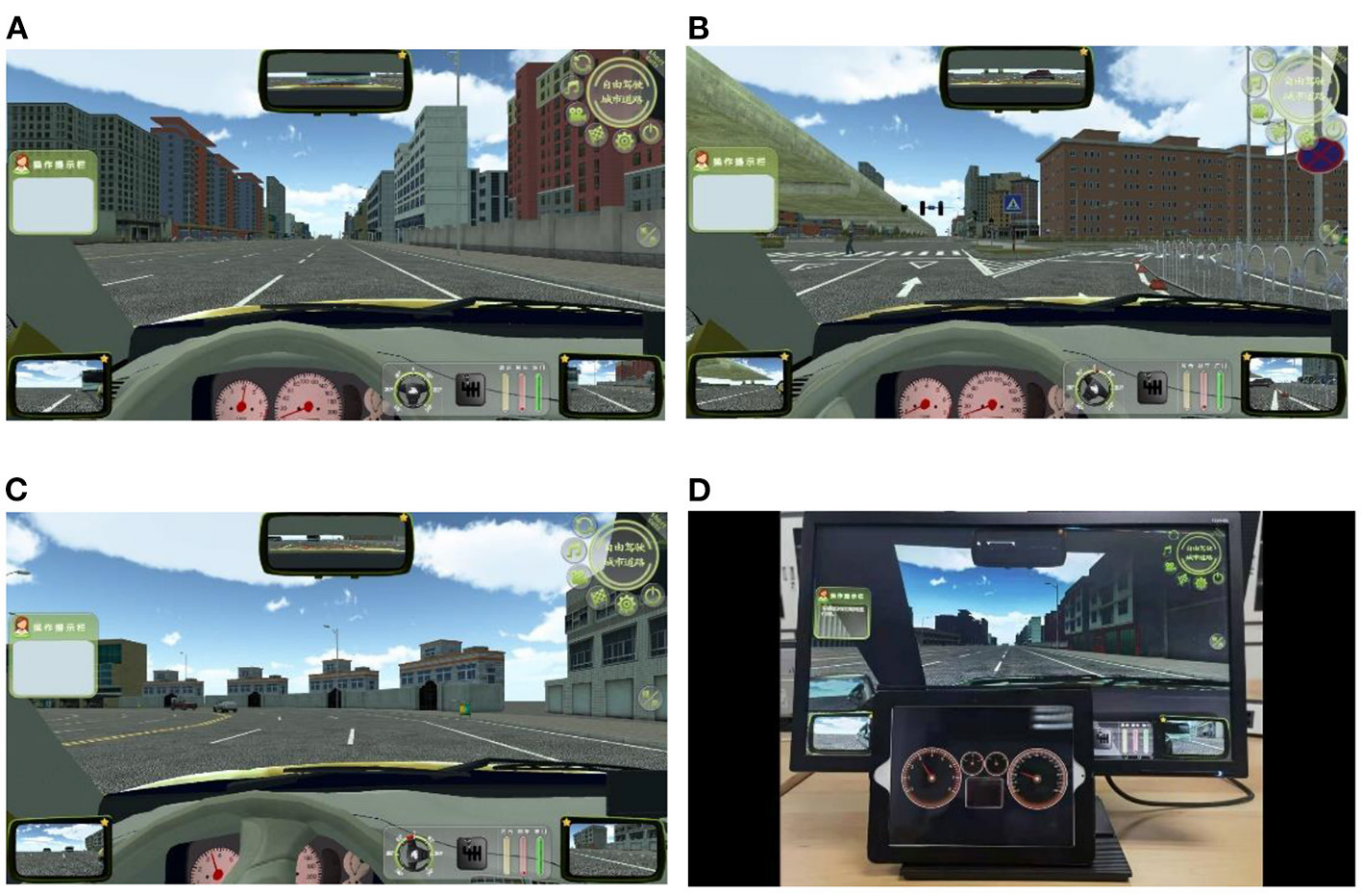
Part of the scenes on the driving simulator screen: **(A)** straight line, **(B)** crosswalk, **(C)** left turning, and **(D)** a photograph of field display.

The experiment was divided into three stages to be carried out (Figure 3):

(1) Before the experiment, the staff explained the test items and precautions to ensure that the subjects understood the purpose and process of the experiment.

(2) At the beginning of the experiment, the subjects needed to accelerate to the speed of 60 km/h [the high speed on ordinary roads in China (44)] and drove at this speed. After that, the staff placed the iPad with the dashboard prototypes in front of the simulator screen and started recording. Each round of driving tasks lasted about 135 s. The driving environment was on urban roads (Figure 4). The driving tasks included: (1) straight line driving; (2) turning left; (3) turning right; (4) shifting gears according to the prompt information; (5) crossing an intersection; (6) crossing a crosswalk; (7) turning around; (8) parking on the side. The subjects needed to perform four rounds of driving tasks totally and the whole experiment lasted about 9 min. Each time the subject completed one round of tasks and returned to the starting point of the route, the dashboard prototype on the iPad would be switched to the next one. In the process of driving, the subjects should observe the prototype according to the voice prompt, and report the reading of the tachometer and speedometer every 20 s. There was no time limit for reading the dashboard so that the subjects should finish reading in their normal cognitive state. If the subjects felt tired, they could apply to terminate the test and start again after a rest so as to eliminate the error caused by fatigue effect.

In the experiment, only the reading of tachometer and speedometer was studied. Other information, such as gear position, navigation prompt, emergency alarm, time, and temperature, were not included. Besides, considering the universality in the context of sharing, the study focused on the functional layout of the dashboards. And personal preference settings such as the font, dynamic effect, visual style of graphics, color, and brightness were taken as controlled variables.

(3) After the experiment, the subjects were required to fill in the system usability scale (SUS) record sheet. Then the staff saved the data and cleaned the test site.

The period from the beginning of scanning to the time when the subject discovered the target dial was the searching time. And the duration from the beginning of reading the instruments to the end of reading was the reading time. The sum of the above two was the total task completion time. Through real-time eye tracking, the three durations could be obtained. And the operant behaviors of the subjects were recorded simultaneously.

Based on the car sharing application situation, the experiment mainly evaluated the interaction efficiency, eye movement characteristics and mental stress changes of the drivers (Table 1). When users accessed a shared car and faced with unfamiliar equipment and environment, the three indicators were important feedback of user experience. In addition, the subjective evaluation was collected by SUS to analyze the relationship between eye movement characteristics of the subjects and usability of the dashboard. The scale being made up of 10 items had gained widespread application in the field of usability research (45). Respondents were asked to score the items with an integer value from 1 to 5 according to their perception after using an interface system (45). The scale was applicable to appraise the HMI of passenger cars (20, 44). Large sample researches on the scale presented a reliability coefficient of 0.91, which showed a good internal consistency reliability (46, 47).

**Table 1:** Dashboard interaction evaluation index system of shared cars.

**The 1st-grade index**	**The 2nd-grade index**	**Description**
Interaction efficiency	Searching time	The period from the beginning of scanning to the time when the subject discovered the target dial
	Reading time	The duration from the beginning of reading the instruments to the end of reading
	Total task completion time	The sum of the searching time and reading time
	Reading accuracy	The ratio of cases being read correctly to the total number of reading times
Eye movement characteristics	Fixation times	The average number of fixation times of the subjects under each dashboard during the reading process
	Fixation duration	The average fixation time of the subjects under each dashboard during the reading process
	Pupil diameter	The average pupil diameter of the subjects under each dashboard during the reading process
Mental stress changes	Heart rate	The frequency of heart contraction (times/min), which was taken every 3s
System usability	SUS score	The subjects assessed the 10 items in the scale and calculated the score.

#### Subjects

Fifty-eight subjects were recruited to participate in the experiment, aged 23–36. The subjects consisted of postgraduates, Ph. D candidates and young teachers who majored in ergonomics or industrial design. All the subjects had more than 3 years of driving experience and were familiar with the information on the digital instruments. All of them had used rental cars and among them, 38 (about 65.5% of the subjects) had rented both basic and high-end cars and could adapt to a variety of dashboard layouts. The car sharing platforms that the subjects had used included GoFun, Yidu, Morefun, CAR (China Auto Rental), EVCARD, Urcar, and GreenGo. Basic information of the subjects is listed in Table 2.

Besides, the recruited subjects ought to meet the following requirements:

(1) They could realize and comprehend the instrument prototypes presented in the experiment;
(2) They were in good health and had no chronic diseases such as cardiopathy or epilepsy;
(3) Their eyesight (wearing glasses) was normal and there was no eye diseases.

**Table 2:** Basic information of the subjects.

**Gender**	***N***	**%**	**Driving experience**	***N***	**%**
Male	31	53.45	3–5 years	39	67.24
Female	27	46.55	5.1–10 years	17	29.31
			More than 10 years	2	3.45
**Age**	***N***	**%**	**Education**	***N***	**%**
23–29 years old	46	79.31	College and University	32	55.17
30–36 years old	12	20.69	Master	18	31.04
			Ph.D	8	13.79

### LSTM Network and the Time Series Characteristics of HR

HR, which means the frequency of heart contraction, is an important factor in cardiac work and one of the important mechanisms of increased cardiac output. HR related indicators could effectively describe the driver's psychological state and mental stress changes (30). In studies of dashboard recognition performance, it is needed to consider the driver's HR variation patterns during the experiment. The driver's ECG is physiological data collected according to the time series, which contains abundant characteristic information in time sequence. In this study, an LSTM network was used to identify and predict the mean HR of the subjects in the process of dashboard interaction, and to judge its time series characteristics.

In researches of regression and prediction of time series data, the Auto-Regressive Integrated Moving Average (ARIMA) model is a classical method, which can better reflect the linear characteristics in time series. However, it is difficult to deal with the non-linear changes of the data fully and effectively by the ARIMA model. In addition, existing researches showed that prediction models based on traditional back propagation (BP) neural network or radial basis function (RBF) neural network could achieve good prediction results of time series, but the robustness was poor (48, 49). An effective method to solve this kind of problem is recurrent neural network (RNN). RNN not only learnt the information of current time, but also relied on the previous sequence information. For example, Vemula et al. used structured RNN to model each individual, and used the spatiotemporal relationship diagram to describe the trajectory change law of each individual with time and space (50). The network was suitable for dealing with ECG signals during driving. But the main problem of traditional RNN was gradient disappearance. LSTM is a form of RNN. The emergence of LSTM solved this problem and could effectively handle and predict the problems of relatively long intervals and delay in time series (51). The model could not only reflect the forward and backward correlation of HR signals, but also dispose of the long-term dependencies problem (41). In the analysis of many time series subjects, such as voice recognition, natural language processing and electroencephalogram (EEG), LSTM network had achieved important results. Because the time characteristics of HR during driving were similar to the above time series information, we applied LSTM to recognize and predict the subjects' average HR after parameter debugging and analyzed the accuracy of the model.

The three gates in the memory cell of LSTM were input gate, forget gate and output gate, respectively. When the HR information of a certain time point was input into the network, the structure of the network determined whether the information was useful. Information that needed to be memorized for a long time was remembered, and unimportant information was forgotten. The learning model of HR changes during a period of reading interaction was a model based on time series and selective memory. Therefore, we used LSTM to train the driver's HR variation.

**Figure 5:**
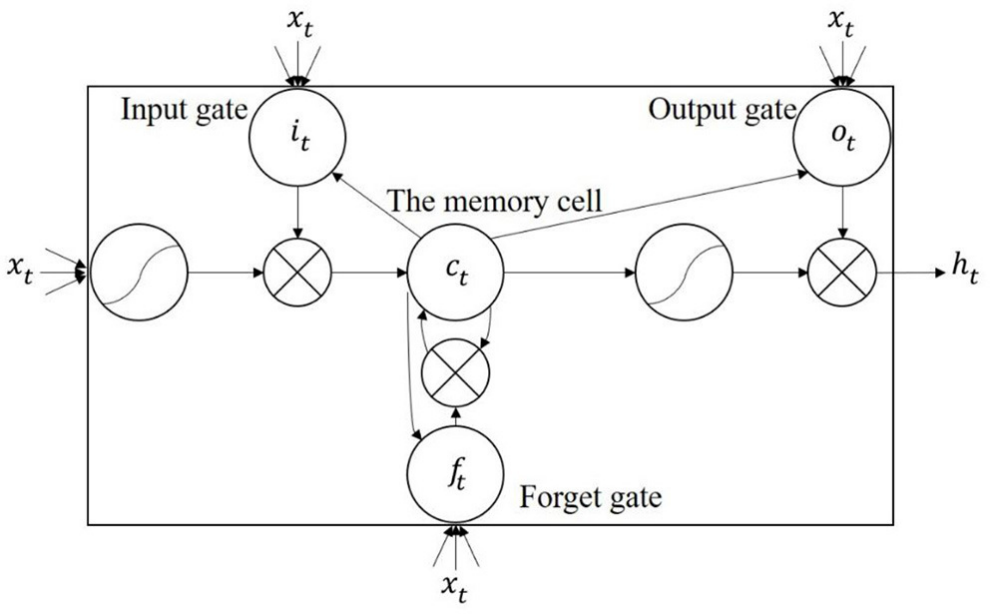
The memory cell of LSTM.

Figure 5 described the memory unit of the LSTM network (52). For the input vector sequence *x* = (*x*_1_, …, *x*_*T*_) in the recurrent neural network, the hidden layer vector sequence *h* = (*h*_1_, …, *h*_*T*_) and the output vector sequence *y* = (*y*_1_, …, *y*_*T*_) were calculated through the iterations of the following formulas from time step 1 to time step T:


(1) $$\begin{array}{l} h_{1}\;=\;\varphi \left(W_{xh}x_{t}\;+\;W_{hh}h_{t\;-\;1}\;+\;b_{h}\right) \end{array}$$

(2) $$\begin{array}{l} y_{t}\;=\;W_{hy}h_{t}\;+\;b_{y} \end{array}$$

where *W* was the weight matrix, for example, *W*_*xh*_ was the weight matrix from the input layer to the hidden layer. *b* was the bias vector, for example, *b*_*h*_ was the hidden layer bias vector. φ was a hidden layer function. The calculation of φ function was realized by the following formulas:


(3) $$\begin{array}{lll} i_{t}\; & = & \;\sigma \left(W_{xi}x_{t}\;+\;W_{hi}h_{t\;-\;1}\;+\;W_{ci}c_{t\;-\;1}\;+\;b_{i}\right) \end{array}$$

(4) $$\begin{array}{lll} f_{t}\; & = & \;\sigma \left(W_{xf}x_{t}\;+\;W_{hf}h_{t\;-\;1}\;+\;W_{cf}c_{t\;-\;1}\;+\;b_{f}\right) \end{array}$$

(5) $$\begin{array}{lll} o_{t}\; & = & \;\sigma \left(W_{xo}x_{t}\;+\;W_{ho}h_{t\;-\;1}\;+\;W_{co}c_{t\;-\;1}\;+\;b_{o}\right) \end{array}$$

(6) $$\begin{array}{lll} c_{t}\; & = & \;f_{t}c_{t\;-\;1}\;+\;i_{t}\text{tanh}\left({\text{W}}_{\text{xc}}{\text{x}}_{\text{t}}\;+\;{\text{W}}_{\text{hc}}{\text{h}}_{\text{t }-\;1}\;+\;{\text{b}}_{\text{c}}\right) \end{array}$$

(7) $$\begin{array}{lll} h_{t}\; & = & \;o_{t}\text{tanh}\left(c_{t}\right) \end{array}$$

where σ was the sigmoid function given by σ(*x*) = (1 +^*e*^−*x*^)−1^. *i*, *f*, *o* and *c* were the input gate, forget gate, output gate and activation vector of the memory cell, respectively, which had the same dimension as the hidden layer vector *h*. The weight matrices from the memory cell to the gates (such as *W*_*ci*_) were diagonal matrices.

The input of LSTM network of the next time point was affected by the output information of the previous moment. The network had prolongment in time series, which could save the timing information forward and backwards, and was no longer restricted by the limitation of traditional neural networks that only stored spatial information. When integrated with ECG signals, LSTM could deeply mine the time correlations among the signal points.

In this experiment, mainly the single layer LSTM structure was applied. The input data of the model was the first to the penultimate HR value of the training set or the testing set, and the output data was the second to the last value. The tanh function was selected as the activation function of the model. The initial learning rate was set to 0.005 and the number of nodes in the hidden layer was 150. In the process of training and recognition, the current state of the hidden layer could be changed by inputting the current information and the state of the previous moment. And this step was cycled continuously until the end of processing. The maximum number of epochs was set to 150.

The loss on the mini-batch was devoted by the following formula:


(8) $$\begin{array}{l} \text{loss }=\;\frac{1}{2\text{N}}\overset{\text{M}}{\underset{\text{i }=\;1}{\sum }}{\left({\text{X}}_{\text{i}}\;-\;{\text{T}}_{\text{i}}\right)}^{2} \end{array}$$

where *X*_*i*_ was the network prediction, *T*_*i*_ was the real value, *M* was the total number of prediction values in *X*, and *N* was the total number of observations in *X*.

### Multinomial Logistic Regression

Binomial and multinomial logit models played an important role in studies of traffic demands and vehicle safety (53, 54). In this experiment, the subjects needed to interact with the four dashboard prototypes to complete the tasks of reading. In order to analyze the relationship between users' interaction efficiency and their HR variation during the interface interaction, an unordered multinomial logistic regression model was introduced in the study. The predicted variable was the type of the dashboard layouts, which could be divided into four categories (Figure 2). Therefore, the logistic regression model was expressed as follows:


(9) $$\begin{array}{l} \ln\left[\frac{p\left(y\;=\;j|x\right)}{p\left(y\;=\;J|x\right)}\right]\;=\;\alpha_{j}+\;\overset{k}{\underset{k\;=\;1}{\sum }}\beta_{jk}x_{k} \end{array}$$

where *j* = 1, 2, 3, 4 was the type of the dashboard layouts, *p*(*y*_*i*_ = *j*) represented the probability that the lowest HR occurred in the interaction process with a certain dashboard in the 4-stage experiment. *x*_*k*_(*k* = 1, 2, 3) meant the *k*th explanatory variable that could predict the type of dashboard. The explanatory variables included average searching time, average reading time and total reading accuracy. β_*jk*_ was the regression coefficient vector of the model. Taking *J* as the reference type, the ratio ($\frac{p\left(y\;=\;j|x\right)}{p\left(y\;=\;J|x\right)}$) of the probability of the lowest HR in the 4-stage experiment occurring on other types of dashboards to the probability of occurring on the type *J* was the odds ratio value (OR). We chose type A dashboard as the reference type, and the following three logistic models were set up:


(10) $$\begin{array}{l} \ln\left(\frac{p_{2}}{p_{1}}\right)\;=\;\alpha_{2}\;+\;\overset{k}{\underset{k\;=\;1}{\sum }}\beta_{2k}x_{k} \end{array}$$

(11) $$\begin{array}{l} \ln\left(\frac{p_{3}}{p_{1}}\right)\;=\;\alpha_{3}\;+\;\overset{k}{\underset{k\;=\;1}{\sum }}\beta_{3k}x_{k} \end{array}$$

(12) $$\begin{array}{l} \ln\left(\frac{p_{4}}{p_{1}}\right)\;=\;\alpha_{4}\;+\;\overset{k}{\underset{k\;=\;1}{\sum }}\beta_{4k}x_{k} \end{array}$$

## Results

### Interaction Features and Usability of the Dashboard Layouts

**Figure 6:**
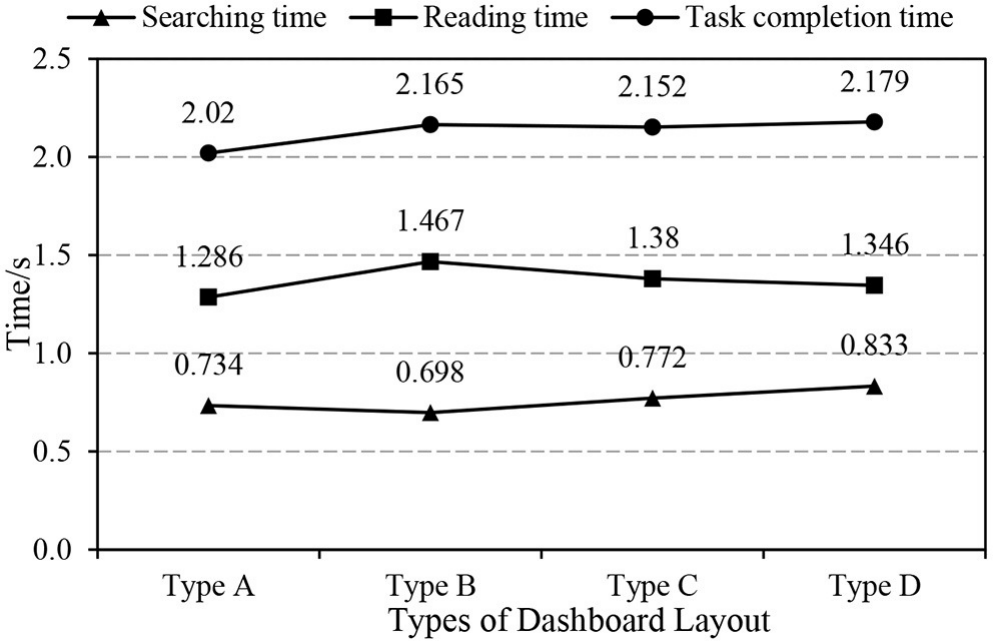
Dashboard reading task completion time vs layouts.

In order to understand the user experience of different dashboard layouts, we analyzed the task completion time under the four layouts. The searching time and reading time under each layout were obtained by averaging the data of the multiple oral reports in each round of reading tasks. The results were shown in Figure 6.

The average searching time and reading time of each type could be used to explore the influencing factors of dashboard recognition efficiency. In the analysis of the differences among the four groups, we chose paired sample *t*-test because the intergroup standard deviation of the two indicators was much larger than the between-group standard deviation. The results showed that the differences in the average searching time and reading time among the dashboards were significant (Tables 3, 4).

**Table 3:** Results of paired sample *t*-test on searching time.

***t*-values**	**Type B**	**Type C**	**Type D**
Type A	2.235*	−2.76**	−7.569***
Type B		−7.755***	−9.118***
Type C			−8.736***

*p < 0.05; **p < 0.01; ***p < 0.001.

**Table 4:** Results of paired sample *t*-test on reading time.

***t*-values**	**Type B**	**Type C**	**Type D**
Type A	−7.161***	−6.42***	−6.555***
Type B		2.536*	3.862***
Type C			2.99**

*p < 0.05; **p < 0.01; ***p < 0.001.

As can be seen from Figure 6, the shortest searching time appeared during the interaction with type B instrument, while the shortest reading time and task completion time occurred in the interaction process of type A, which reflected that the attention level and usability of a certain instrument were not completely consistent. The completion time of a reading task started from the beginning of scanning. And vision played an important role in the accomplishment of the task. Therefore, we made statistics on eye movement data and SUS scores under the four instruments. The results were shown in Table 5.

**Table 5:** The eye movement characteristics and SUS scores under the four dashboard layouts.

**Variables (*n* = 58)**	**Mean**	**Std. Deviation**	**Minimum**	**Maximum**
A_FD (s)	1.029	0.131	0.704	1.243
A_FT (times)	7.766	1.567	4.116	11.285
A_PD (mm)	3.193	0.577	1.836	4.466
A_SUS	84.612	3.529	77.5	92.5
B_FD (s)	1.129	0.133	0.818	1.377
B_FT (times)	7.364	1.197	4.886	10.59
B_PD (mm)	3.56	0.42	2.81	4.572
B_SUS	81.595	3.796	75	90
C_FD (s)	1.378	0.18	0.977	1.739
C_FT (times)	8.029	1.819	3.871	12.132
C_PD (mm)	4.025	0.351	3.233	4.79
C_SUS	78.578	4.192	67.5	87.5
D_FD (s)	1.061	0.098	0.835	1.284
D_FT (times)	8.06	1.065	5.426	9.877
D_PD (mm)	3.828	0.512	2.87	5.199
D_SUS	80.862	3.161	72.5	87.5

Taking the fixation duration (FD), fixation times (FT), and pupil diameter (PD) of each instrument as independent variables and SUS score as the dependent variable, four regression models could be established as follows:


(13) $$\begin{array}{lll} SUS_{A}\; & = & \;7.128X_{1A}\;+\;1.03X_{2A}\;-\;1.821X_{3A} \end{array}$$

(14) $$\begin{array}{lll} SUS_{B}\; & = & \;8.358X_{1B}\;+\;0.549X_{2B}\;-\;3.873X_{3B} \end{array}$$

(15) $$\begin{array}{lll} SUS_{C}\; & = & \;-3.42X_{1C}\;+\;0.802X_{2C}\;-\;3.571X_{3C} \end{array}$$

(16) $$\begin{array}{lll} SUS_{D}\; & = & \;10.476X_{1D}\;-\;0.359X_{2D}\;-\;2.068X_{3D} \end{array}$$

The results of significance tests showed that most eye movement indicators were related to system usability of the dashboards. Except for FT of type B and D instruments (*X*_2*B*_ and *X*_2*D*_) and FD of type C (*X*_1*C*_), other indicators had significant effects on the SUS score of corresponding instruments (*p* < 0.05). Among them, FD and FT both had positive effects on the usability of A-type dashboard. In addition, for type B and D, FD had a positive effect. And for type C, the effect of FT was positive. In contrast, PD had a significant negative effect on the SUS scores of all the four instruments, indicating that during the reading process, the smaller the PD got, the higher the dashboard's usability was. In conclusion, the system usability of the dashboards was in connection with visual ergonomics. And among the four dashboard layouts, the SUS score of type A was the highest (A_SUS = 84.612), while that of type C was the lowest (C_SUS = 78.578).

### Prediction Results of HR Change

**Figure 7:**
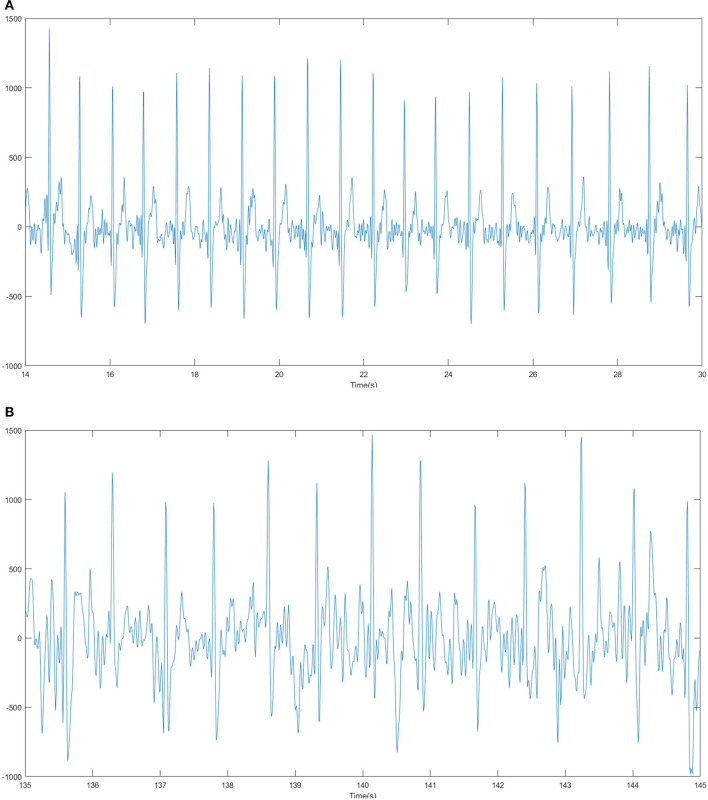
The ECG waveforms of a subject during driving **(A)** in a straight section of the road and **(B)** turning along a large radius curve.

In addition to task completion time and system usability, the change of drivers' mental stress was also an important indicator to further reflect the user experience brought by different dashboard layouts. In this study, the subjects' ECG signals in instrument reading tasks were analyzed. Time domain signals were collected in the experiment. In Figure 7, the ECG waveforms (a) of one of the subjects during driving in a straight section of the road (from the 14th to 30th second of the experiment) and the waveforms (b) while turning along a large radius curve (in the period from the 135th to 145th second) were illustrated.

An LSTM model was established to predict HR. Eighty percentage of all the HR values were taken as the training set and the remaining data as the testing set. After analyzing the change curve of root mean squared error (RMSE) and the training loss curve of the training process, it could be seen that after 80 cycles of iterations, the above two curves entered into a convergence state, and the values of the loss function were stable near the minimum value. It meant that the network could also accommodate a larger scale of drivers' HR data and recognize the features, which reflected a good potential for large-scale database recognition. On the other hand, it also indicated that the network was not likely to produce gradient explosion or vanishing, and had a good stability.

**Figure 8:**
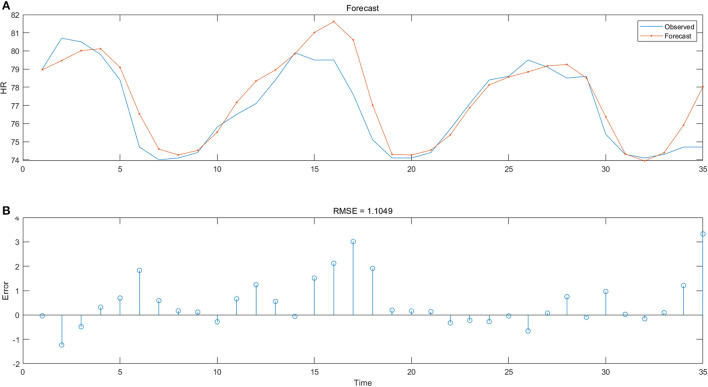
**(A)** HR prediction results and **(B)** RMSE changes.

Figure 8A revealed the gap between the real values of the testing set (the blue curve) and the predicted values of the model (the orange curve). The horizontal axis represented the number of samples in the testing set, and the vertical axis meant the HR values of the samples. Figure 8B showed the change of errors generated in each prediction. It could be seen that the trend of predicted values of this model was basically consistent with that of real values.

For HR recognition during a driving process with only 9 min, the prediction accuracy of this model (RMSE = 1.105, MAE = 0.009) was acceptable, which reflected that the LSTM model could better fit the non-linear, abrupt and periodic laws of HR data. Hence one could see that the model was more suitable for the prediction and analysis of HR during driving. Due to the short experimental time, the size of the collected data was small. And only 58 subjects' HR data were included in the network. If the scale of the training set was expanded in practical application, the prediction accuracy should be improved.

In the experiment, the average HR of the subjects fluctuated regularly with time, which showed that the ECG during a driving task would change with the time going on and the subjects' attention switching between the HMI and the road. This series of HR variation when reading the instruments was consistent with the subjects' nervous mood. Literature (55) also discovered that when the driver was in the emotional situation of appreciation or mental focus (during simple tasks), the HR would show fluctuations similar to Figure 8A. The HR during the interaction with a dashboard presented a periodic pattern rather than a continuous increase or decrease. Therefore, it was unreasonable to evaluate the quality of an interface design and its level of information transmission only by the users' HR variation. It was also necessary to consider the influence of searching time and reading time on the HR.

### Influencing Factors of the Occurrence Probability of Minimum HR

After understanding the time characteristics of HR in the process of driving and instrument reading, we used logistic regression models to analyze the influencing factors of the probability of the lowest HR occurring during the interaction with a certain instrument layout. The results were shown in Table 6.

**Table 6:** Results of the logistic regression.

		**Coef**.	**Std. Err**.	***z***	***p* > |z|**
Type A		(base outcome)			
Type B	Searching time	−3.68	1.448	−2.54	0.011
	Reading time	2.223	1.112	2	0.046
	Reading accuracy	−3.016	2.745	−1.1	0.272
	_cons	0.015	2.243	0.01	0.995
Type C	Searching time	5.248	2.124	2.47	0.013
	Reading time	−3.64	1.526	−2.39	0.017
	Reading accuracy	−5.022	3.374	−1.49	0.137
	_cons	2.91	2.818	1.03	0.302
Type D	Searching time	6.967	2.63	2.65	0.008
	Reading time	−5.963	1.97	−3.03	0.002
	Reading accuracy	−8.848	4.414	−2	0.045
	_cons	5.686	3.163	1.8	0.072

Pseudo *R*^2^ of the model was 0.453. Besides, an likelihood ratio test was done and the chi-square value was 68.08 (*p* < 0.001), indicating that the overall fitting effect of the model was good. It could be seen that compared with type A, the subjects' searching time and reading time when interacting with type B and C instruments presented a significant influence on the dependent variable, while the total reading accuracy had no significant impact. Nevertheless, in the reading process of type D, the searching time, reading time and reading accuracy all had a significant effect on the dependent variable.

To sum up, in the process of instrument reading interaction, which kind of interface layout the lowest mental stress occurred on could be explained by these three variables to a certain extent. The users' average searching time, reading time and reading accuracy brought about by different layouts were important for the context of car sharing, and needed to be taken into consideration in usability tests of dashboard design schemes.

## Discussion

For any interface design, a reasonable information architecture is a key factor to build user experience. When it comes to automobile digital instrument panels, the layouts should be convenient for drivers to quickly identify and organize a variety of information types. According to the existing literatures, at present, researchers tended to study the shape and character encoding of instrument panels (14), or different properties of the gauges, such as the form (circle or linear), indicator styles (pointers or bar graphs) and direction (horizontal or vertical) (5). However, there is a shortage of researches for a specific layout. This will lead to an unclear effect of information architecture and inadequate research on dashboard interaction. A dashboard with a clear display layout design should methodically feed back the information to the tenants who do not know the vehicle very well.

In this study, from the perspective of recognition efficiency in human-computer interaction, we found a suitable dashboard layout for shared cars. The results showed that when users accessed unfamiliar dashboards, different layouts would bring about significant distinctions in interactive performance. Besides, the HR during driving and reading the instruments presented a certain periodicity, which should be considered in design works for shared cars. Currently, the particularity of user experience in shared cars compared with that in private cars was a hot issue. For example, the stated preference (SP) approach (56) was frequently used to determine the characteristics of car-sharing users. The usability and ease of use of human-computer interaction was very important for shared car users when being transmitted to a specific vehicle. Kuemmerling et al. had studied the efficiency of human-vehicle-interfaces in shared vehicles when setting driver assistant functions on the dashboard, so as to improve the safety, comfort and usability (3). This study further clarified the problems about instrument layouts that shared cars should pay attention to when considering dashboard recognition.

Regression models were established to explore the effects of fixation duration, fixation times and pupil diameter on the usability of the four dashboard layouts. The results showed that the usability of a dashboard system had a bearing on visual ergonomics, which was consistent with existing research conclusions. Some eye movement characteristics during driving, such as fixations and glances, could reflect the driver's workload (44) and information demands under a certain dashboard, which related to in-vehicle display and traffic light situation (57). Therefore, the study further analyzed the impact of visual task completion time and reading efficiency on drivers' mental stress under various dashboard layouts, which provided a theoretical basis for HMI design of shared cars.

In order to probe the drivers' mental stress changes, we analyzed the HR data. Although some studies had traditionally used feedforward neural networks such as Back Propagation Neural Networks to predict HR (55), which could also get good accuracy, this kind of networks had no connection among nodes, and could only sense the data fluctuation in a very short time in time series, or adopted the same time-varying weights. ECG signals were collected according to time series. Feedforward networks could not fully learn the non-linear trend of ECG with time, which limited the performance of the models. The study indicated that constructing an LSTM network was an effective way to solve this problem. The results showed that using the LSTM model to mine the strong correlations among HR data points and extract the time characteristics could improve the prediction effect of HR in terms of algorithm.

Existing researches showed that HR was significantly affected by speed [*F*_(3, 184)_ = 43.076; *p* < 0.001], but not by interface [*F*_(1, 184)_ = 1.726; *p* = 0.191] (44). However, this study found that the drivers' HR in the process of interface interaction presented characteristics in time series. Thus, it was necessary to take the influence of interaction features on HR brought by different interfaces into consideration. The study indicated that type A layout was the most conducive to the overall reading of an instrument to some extent because the searching time and reading time under this layout were both the shortest. And compared with type A instruments, the shorter the searching time under type B was, or the shorter the reading time under type C and D was, the more drivers would present lower HR. This meant that the differences in task completion time caused by the dashboard layout design had an impact on drivers' mental stress. Existing researches had also shown that when drivers interacted with a cockpit, the panel components would influence the quality of information delivery and the accomplishment of visual tasks (58, 59). Meanwhile, in design works of layouts, it was inappropriate to pursue a shorter time only. For example, compared with type A layout, a lower HR under type D was accompanied by a lower reading accuracy (*p* < 0.05), which was not good for driving safety. Therefore, for dashboard layout design, it was necessary to consider both reading time and reading accuracy according to the results in Table 6.

Entropy weight method can be used in future research. Based on the characteristics of the tachometer and speedometer studied in this study, the indicators of other dashboard information under the four layouts, such as gear position, navigation prompt, emergency alarm, time and temperature, can be further supplemented. Through the original data matrix and the entropy weight of each indicator, the comprehensive scores of the layouts can be calculated, so as to sort the importance of each layout. Based on the optimal scheme, visual elements such as color, texture and dynamic effect can be added, which will help to achieve a reasonable interface design that is more in line with the driver's eye movement and favorable to adjusting their heart rate.

Although the specific implementation of car sharing varied with business models, these implementation schemes shared common goals in reducing total travel volume and distance, diversifying travel modes and improving efficiency (60, 61). When consumers decided to adopt peer-to-peer car sharing services instead of business-to-customer services, public concerns about sustainable solutions would play a certain role (62). However, the willingness of current consumers to continue using shared cars was not high. By optimizing the user experience, this willingness could be strengthened to contribute to the promotion of shared cars. In this way the green level of the urban environment and sustainable utilization of resources could be improved.

## Limitation

One limitation came from the immersion degree of the experiment. In order to reduce the subjects' sense of strangeness, the simulator we chose was a simple one commonly used in driving school training. The simulator and display screen were separate and not integrated into one workspace, which led to a lower immersion. In the experiment, the subjects had a subconscious feeling that they were observed and was more tense than driving in normal times. In future, we plan to use an integrated driving simulator to carry out experiments with a higher immersion degree, and collect ECG signals in a more natural state. Besides, in the process we found that the total experiment duration about 9 min was a little short. Some subjects failed to fully enter the natural driving state, while the experiment had been over. In order to get truer HR data, the experiment time should be lengthened.

Another shortcoming existed in the limited sample size. It took a long time to train the LSTM model, which prevented us from testing all possible parameters. Due to the complexity of driving behaviors, for the sake of improving the robustness of the network, a dataset provided by a larger scale of subjects would be better. What's more, speed was taken as a controlled variable in this experiment, so the differences of HR and eye movement characteristics when reading instruments at various speed were not explored. In next phase, the speed would be taken into the study to analyze the influence of different speed on instrument recognition.

## Conclusions

In this study, we reported an experiment about dashboard recognition of 58 subjects, which compared the reading efficiency, system usability and drivers' mental stress of four typical dashboard layouts under the context of car sharing. The main conclusions were as follows:

(1) A statistical analysis presented that among the four types of dashboards, the reading time and task completion time of type A were the shortest, but the searching time was longer than that of type B. Besides, the average fixation time of type A was the shortest (Mean = 1.029), and the system usability of this type was the highest correspondingly (SUS = 84.612). On the other side, the average fixation times of type B was the lowest (Mean = 7.364). By linear regression models, it could be found that most of the eye movement indicators under the four dashboards had significant impacts on system usability. Therefore, optimizing the visual ergonomics of a dashboard through the interface layout design helps to improve the usability of the system. And it will affect the task completion time, and raise the efficiency of reading.

(2) The prediction effect of the LSTM model was good with an RMSE = 1.105 and an MAE = 0.009. It confirmed that the network could effectively fit the non-linear, abrupt and periodic laws of HR data after parameter debugging. Better prediction performance was achieved from the perspective of algorithm, which contributed to analyzing the HR changes in the process of dashboard interaction.

(3) Multinomial logistic regression models indicated that the searching time, reading time and reading accuracy could explain what kind of dashboard layout a low mental stress was prone to occur on. For example, compared with type A layout, the higher the reading accuracy under type D was, the higher the HR would be (p < 0.05). Thus, in design works for shared car dashboard layouts, it was unreasonable to appraise the interaction performance of a design scheme only by temporal indicators such as searching time and reading time. The reading accuracy was also crucial.

## Data Availability Statement

The raw data supporting the conclusions of this article will be made available by the authors. If the subjects and the authors verify and approve the purpose of the requester, it can be provided.

## Ethics Statement

The studies involving human participants were reviewed and approved by North China University of Technology. The patients/participants provided their written informed consent to participate in this study.

## Author Contributions

HY: conceptualization, data curation, formal analysis, investigation, methodology, and roles/writing—original draft. YW: funding acquisition, project administration, resources, software, supervision, and validation. RJ: visualization and writing—review and editing. All authors contributed to the article and approved the submitted version.

## Funding

This research was funded by Beijing Urban Governance Research Project, Grant Number: 21CSZL09; Scientific Research Program of Beijing Education Commission, Grant Number: KM202010009003; and Chinese Ergonomics Society & Kingfar Joint Research Fund for Outstanding Young Scholars, Grant Number: CES-Kingfar-2019-001.

## Conflict of Interest

The authors declare that the research was conducted in the absence of any commercial or financial relationships that could be construed as a potential conflict of interest.

## Publisher's Note

All claims expressed in this article are solely those of the authors and do not necessarily represent those of their affiliated organizations, or those of the publisher, the editors and the reviewers. Any product that may be evaluated in this article, or claim that may be made by its manufacturer, is not guaranteed or endorsed by the publisher.
